# Health-Related Quality of Life in Lung Cancer Survivors: Sociodemographic, Clinical, and Psychosocial Modulators

**DOI:** 10.3390/curroncol33070428

**Published:** 2026-07-17

**Authors:** Yolanda Andreu, Ana Soto-Rubio, Beatriz Gil-Juliá, Carmen Picazo, Inmaculada Maestu, Silvia Fernández

**Affiliations:** 1 Department of Personality, Assessment, and Psychological Treatments, Faculty of Psychology and Speech Therapy, University of Valencia, 46021 Valencia, Spain; yolanda.andreu@uv.es (Y.A.); beatriz.gil@uv.es (B.G.-J.); 2 Department of Psychology and Sociology, Faculty of Social and Human Sciences of Teruel, University of Zaragoza, 44003 Teruel, Spain; carmen.picazo@unizar.es; 3 Department of Oncology, Doctor Peset University Hospital, 46017 Valencia, Spain; inmaculada.c.maestu@uv.es; 4 Research Group INCUE, Valencia Health Department, Doctor Peset University Hospital, 46017 Valencia, Spain; silviafp9@gmail.com; 5 Carena Association of Psycho-Oncology, Valencia Health Department, Doctor Peset University Hospital, 46017 Valencia, Spain

**Keywords:** lung cancer survivors, quality of life, association between HRQOL and distress, quality of life modulators, clinical, sociodemographic, psychosocial factors

## Abstract

The study aims to investigate the Health-Related Quality of Life (HRQOL) in lung cancer survivors, comparing it with the HRQOL of survivors of other types of cancer, analyzing its association with clinically significant distress, and exploring the modulating role of sociodemographic, clinical, and psychosocial variables. A total of 141 lung cancer survivors who had completed treatment with curative intent and were disease-free completed the Quality of Life in Adult Cancer Survivors questionnaire (QLACS), the Brief Symptom Inventory-18 (BSI-18), the Medical Outcomes Study–Social Support Survey (MOS-SSS), and the Utrecht Proactive Coping Competence scale (UPCC). Several multivariate analyses of variance (MANOVA) were performed to address the study objectives. Statistical analysis was performed using IBM SPSS Statistics 22.0. The overall HRQOL of lung cancer survivors did not differ from the HRQOL of hematologic, breast, and gynaecologic cancer survivors and was lower than that of colorectal, head/neck, prostate, and melanoma cancer survivors. HRQOL was associated with clinically significant distress. Younger age, female sex, lower levels of proactive coping, and less positive social interaction were independently associated with worse HRQOL in lung cancer survivors. The overall HRQOL of lung cancer survivors is among those with the poorest HRQOL compared with other cancer survivors’ groups. The modifiable nature of the psychosocial variables that characterize the risk profile (with the exception of sociodemographic ones) allows for the establishment of more ambitious goals than the simple establishment of subgroups on which to prioritize care. The team of professionals involved in the care of lung cancer survivors should also provide intervention strategies that improve their well-being.

## Simple Summary

This study investigated the Health-Related Quality of Life (HRQOL) of lung cancer survivors, comparing it with that of survivors of other cancers and examining the role of sociodemographic, clinical, and psychosocial variables. A total of 141 disease-free lung cancer survivors who had completed treatment with curative intent participated. Overall HRQOL was similar to that of hematologic, breast, and gynaecologic cancer survivors, but lower than that of colorectal, head and neck, prostate, and melanoma cancer survivors. Younger age, female sex, clinically significant distress, low proactive coping, and low social support were associated with worse HRQOL. Since most factors associated with poorer HRQOL are modifiable, healthcare professionals should not only identify high-risk groups, but also provide interventions to improve survivors’ well-being. Professional teams involved in the care of lung cancer survivors should provide intervention strategies that improve their well-being.

## 1. Introduction

Cancer survivors often experience late and long-term effects of cancer and its treatment, facing a variety of challenges that affect their overall health and well-being, including physical [1,2], emotional [3,4], and social [3,5,6] dimensions. In comparison with other types of cancer and given that it affects a vital organ, lung cancer (LC) is associated with a greater burden of symptoms, greater deterioration in overall health status [7], and more frequent and often more severe deterioration in health-related quality of life (HRQOL) [8]. In addition to the fact that some of the common side effects of cancer treatments, such as fatigue and pain, may be more severe in LC and even worsen over time, LC is associated with a specific cluster of symptoms that includes cough, loss of appetite, and shortness of breath [9,10,11].

Possibly due to the historically low survival rates of LC, its inclusion in HRQOL studies in cancer survivors has been rare. However, over the past two decades, advances in understanding tumor biology and the development of new therapies, including immunotherapy and targeted therapy, along with the introduction of screening, have led to an increase in five-year survival [12,13]. This increase in survival makes understanding HRQOL in this understudied subgroup of survivors a research priority.

The provision of optimal and comprehensive healthcare and the establishment of personalized care pathways for cancer survivors, as recommended by the National Comprehensive Cancer Network [14] in its survival guidelines, is hindered by our still limited knowledge of HRQOL in LC survivors. It is therefore essential to conduct research that sheds light on the existing deficits in the HRQOL of LC survivors, while identifying possible modulating factors that allow for the establishment of risk subgroups on which to prioritize care.

The available studies in lung cancer survivors are scarce and, even more than those conducted on other cancer types, are affected by a limitation that has long characterized research in cancer survivorship: the inclusion of heterogeneous groups in terms of clinical phase and disease status [15]. In fact, based on their poor prognosis, studies addressing short survival in LC argue that survival in this type of cancer can be defined as one year after diagnosis [9,16,17] and/or assume a generic definition of survivor that begins at the time of diagnosis [13,18]. Based on such conceptualizations of survival, it is common to conduct a joint analysis of heterogeneous groups of participants in phases both inside and outside of cancer treatment and both disease-free and with metastasis/recurrence.

Existing studies to date agree in highlighting the significant deterioration in HRQOL in LC survivors [13,19,20]. Regarding the variables that modulate HRQOL, it should be noted that relevant psychosocial variables have not been explored and that there are contradictory results regarding demographic and clinical variables. Thus, for example, we lack results on LC survival with respect to two variables that are frequently explored in survival rates for other types of cancer and that have been shown to play a significant role [21], namely coping and social support. At the same time, we find widely contradictory results regarding the modulating role of two variables that have been extensively explored in other types of cancer, such as treatment and sex. In the first case, some studies support its association with HRQOL [22,23], while others do not [8,9]. With regard to sex, the results have ranged from supporting its non-association with the HRQOL of LC survivors [9,24] to supporting poorer HRQOL in males [25] and also in females [26,27]. Consequently, the existing results do not allow firm conclusions to be drawn regarding the HRQOL that characterizes LC survivors, how this HRQOL compares to that of survivors of other types of cancer, or what its sociodemographic, clinical, psychological, and social predictors are. Therefore, further studies are needed to provide answers to these questions.

In line with our previous work [15,28] and with the twofold objective of reducing the source of variability associated with the lack of a universally accepted definition of “cancer survivor” [29,30] and increasing the comparability of the results obtained, we believe that improved survival rates allow us to adopt a definition of LC survivor that is more consistent with the concept of survival as a phase of cancer control. In this regard, we define “cancer survivor” as a person who has completed primary cancer treatment with curative intent and is free of disease [31,32,33].

Our objective in this study is to explore HRQOL in LC survivors (i) by comparing it with that reported in a previous study by survivors (defined as previously indicated, belonging to the same cohort and recruited using the same procedure) of seven other types of cancer (hematological, breast, gynecological, colorectal, head/neck, melanoma, and prostate), (ii) exploring its association with clinically significant distress, and (iii) analyzing possible sociodemographic (age, sex, marital status, employment status), clinical (primary treatment, survival phase), psychological (distress and coping), and social (social support) modulators of HRQOL.

## 2. Materials and Methods

### 2.1. Procedure and Participants

This cross-sectional study is part of a research project on QoL and unmet psychosocial needs in adult oncology survivors approved by the Ethic Committee of the different participating medical institutions and cancer patient associations. A total 178 LC survivors were approached. Eligibility criteria included (i) age ≥ 18 years at diagnosis, (ii) being disease-free (no evidence of disease), and (iii) completion of treatment with curative intent at least 1 month prior to enrollment. Of the 178 approached people, 141 (79%) were eligible and provided their signed written consent. A psychologist carried out the assessment during one of the survivors’ visit to the health centres. The final sample included survivors ranging in age from 23 to 90 years (M = 63.7; SD = 11.0), two-thirds of whom were men (66.7%). Most of the participants (74.3%) were married or living with a partner, were not employed (74.8%) and had received systemic treatment (81.6%). The mean length of time since completion of primary treatment was 3 years (range: 1 month–28 years): 31.3% had completed it in the previous 12 months (re-entry survivorship phase, RES), 25% had completed it at least 5 years before the moment of interview (long-term survivorship phase, LTS), and 43.4% had exceeded 12 months after primary treatment but had not yet reached 5 years (early survivorship phase, ES) (Table 1).

### 2.2. Measures and Instruments

Health-related QoL (HRQOL). The Quality of Life in Adult Cancer Survivors (QLACS) [34], Spanish version [35], comprises 47 items grouped into twelve domains (negative feelings, positive feelings, cognitive problems, physical pain, problems with sexual functioning, fatigue, social avoidance, financial problems, family-related distress, appearance concerns, distress over recurrence, and benefits of cancer. The QLACS assesses Health-Related Quality of Life (HRQOL) in the past month on a seven-point Likert scale (1 = never trough 7 = always), with higher scores indicating lower HRQOL (except for positive feelings and benefits). In addition to the score in each of the twelve domains, it is also possible to obtain a total score on the scale [36], which excludes the benefits domain. For this reason, this subscale was not considered in this study. Cronbach’s alpha coefficients for both the total score (α = 0.93) and the different domains of the QLACS in this study (range α = 0.72–0.90) were satisfactory.

Emotional Distress. The Brief Symptom Inventory-18 (BSI-18) [37] is a self-reported symptom checklist comprising 18 items rated on a five-point Likert scale (1 = none through 4 = a lot). The respondents are asked to rate how they felt during the previous week. In addition to providing three symptom scores (anxiety, depression, and somatization) and a total score, the BSI-18 allows clinically significant distress to be identified. To do this, the scores were transformed into T scores following sex-specific normative data (a T score ≥ 63 on the GSI or on at least two subscales was classified as a case). The Spanish version of BSI-18 has shown adequate psychometric properties in cancer settings [38]. In our study, Cronbach’s alpha coefficient for the GSI was 0.94.

Proactive Competencies. The Utrecht Proactive Coping Competence scale (UPCC) [39] is a 21-item self-administered questionnaire scored on a 5-point Likert scale ranging from 1 (not at all) to 5 (very much). It was developed to measure competencies in skills associated with proactive coping: a type of future-oriented coping that refers to efforts made to prevent/modify a potentially stressful event before it occurs [40]. Proactive coping is expected to lead to better adaptation to chronic illness, as it allows patients to prevent and prepare in advance for the consequences of the illness [41]. A higher score on the scale indicates higher levels of perceived proactive coping skills. Cronbach’s alpha coefficient was satisfactory (α = 0.97) in the current study.

Perceived social support. The Medical Outcomes Study–Social Support Survey (MOS-SSS) [42], Spanish version [43], is a 19-item self-administered scale, originally designed to assess functional support in a community sample of patients with chronic illness. The items are scored on a 5-point Likert scale and assess the perceived availability of five types of social support: emotional (positive affect, empathetic understanding, and encouragement of expressions of feelings), informational (advice, information, guidance or feedback), tangible (material aid or behavioral assistance), positive social interaction (PSI; availability of others for enjoyable activities), and affective (expressions of love and affect). The sum of all the items also provides a total score on the scale. The Spanish version of MOS-SSS has shown adequate psychometric properties in cancer setting [44]. Cronbach’s alpha coefficients were satisfactory for total scores (α = 0.97) and subscales (range α = 0.88–0.91) in the current study.

### 2.3. Data Analysis

Descriptive statistics were calculated to summarize sociodemographic, cancer-related and psychosocial data. Several multivariate analyses of variance (MANOVA) were performed to address the study objectives. A first multivariate analysis of variance (MANOVA) explored the differences between the HRQOL of the participants in this study and that reported by survivors of different types of cancer in a previous study [15]. Several subsequent MANOVAS and correlational analysis, depending on whether the variable was discrete or continuous tested the association between HRQOL and clinically significant distress (from now on, distress), as well as the possible sociodemographic, clinical, psychological, and social modulators of HRQOL in LC survivors. Due to the small size of some of the resulting subgroups, when possible, as in the case of the age variable, the categories were regrouped (18–64 years and ≥65 years). Finally, a regression analysis explored the independent predictors of HRQOL in lung cancer survivors. The statistical significance level for analyses was *p* ≤ 0.05. Statistical analysis was performed using IBM SPSS Statistics, version 22.0.

## 3. Results

QoL in the lung cancer survivors. Table 2 shows the HRQOL of LC survivors who participated in this study and compares it with that reported by survivors of other types of cancer: hematologic, breast, gynecologic, colorectal, head/neck, prostate, and melanoma, in the aforementioned study [15]. Overall, the results show that the total HRQOL of LC survivors is worse than that of colorectal, head/neck, melanoma, and prostate survivors, while it does not differ from that of hematological, breast, and gynecological survivors. In terms of domains, LC survivors experience more negative feelings, appearance issues, and distress about recurrence than the prostate subgroup; more pain and fatigue than prostate and melanoma survivors; and more distress about family than breast and colorectal survivors. On the other hand, they also show differences in terms of domain with respect to the hematological, breast, and gynecological subgroups: fewer negative feelings, sexual and economic problems than the hematological subgroup, fewer cognitive problems than the hematological and breast subgroups, fewer problems with appearance than the hematological, breast and gynecological subgroups, and less pain than the breast subgroup.

Association between HRQOL and distress. Survivors experiencing distress showed lower average HRQOL and lower HRQOL in each of the domains assessed by the QLACS than participants without distress (Table 3).

Sociodemographic variables and HRQOL. The results of the MANOVAS analyses examining the association between these variables and HRQOL among LC survivors showed no significant association with either marital status (Pillai’s Trace = 0.068, F = 0.84; df hypothesis = 11.00; df error = 125.00; *p* = 0.605) or employment status (Pillai’s Trace = 0.069, F = 0.833; df hypothesis = 11.00; df error = 124.00; *p* = 0.607). Only age and sex were associated with HRQOL (Table 3). Younger age was associated with lower overall HRQOL and, by domain, more negative feelings, cognitive problems, pain, concern about appearance, distress about recurrence, and financial problems than older age. Females reported a lower overall HRQOL and, by domain, more negative feelings, cognitive problems, fatigue, concern about appearance, and distress about recurrence than males.

Clinical variables and HRQOL. Neither the type of primary treatment received (Pillai’s Trace = 0.079, F = 0.988; df hypothesis = 11.00; df error = 126.00; *p* = 0.461) nor survival phase (Pillai’s Trace = 0.191, F = 1.09; df hypothesis = 22.00; df error = 228.00; *p* = 0.354) was associated with HRQOL (Table 3).

Psychosocial variables and HRQOL. Greater proactive coping and greater overall social support were associated with better overall HRQOL and, by domain, with fewer negative feelings, more positive feelings, fewer cognitive problems, and less fatigue (Table 4). In addition, greater proactive coping was also associated with less social avoidance, and greater overall social support was associated with fewer financial problems. With regard to the types of support, the results provided evidence of associations in the expected direction between emotional and informational support, PSI, and affective support, and overall HRQOL; and, by domain, with negative feelings, positive feelings, cognitive problems, financial problems, and (with the exception of affective coping) fatigue. We also found an association between PSI and pain and social avoidance, between affective support and pain, and between tangible support and sexual and financial problems.

Independent predictors of HRQOL. The high correlations (Table 4) between the different types of social support (range: 0.66–0.93) made it inadvisable to include these variables together in a regression analysis. In fact, diagnostic tests indicated that three of the five support types had a variance inflation factor exceeding 5 (FIV range: 2.66–10.59) indicating problematic multicollinearity [45]. Given its association with a greater number of HRQOL dimensions—including total social support—only PSI was included in the regression analysis.

The results of the regression analysis (Table 5) showed that the four variables considered (age, sex, PCI, and PSI) were independent predictors of overall HRQOL (R^2^ = 0.20). No regression analyses were performed for the QLACS subscales of sexual problems and family distress, since none of the variables examined showed a significant association with them. Analysis was also ruled out for the pain subscale, which was associated only with PSI (R^2^ = 0.05). The independent predictors of concerns about appearance and distress—recurrence were exclusively sociodemographic (age and sex; R^2^ = 0.15, and age; R^2^ = 0.09, respectively). Positive feelings and social avoidance were predicted exclusively by psychosocial variables (PCI and PSI; R^2^ = 0.23, and PCI and PSI; R^2^ = 0.10, respectively). Finally, a combination of sociodemographic and psychosocial variables predicted cognitive problems (age, sex, PCI, PSI; R^2^= 0.13), negative feelings (age, proactive coping, and PSI; R^2^ = 0.14), financial problems (age and PSI; R^2^ = 0.11), and fatigue (sex and PSI; R^2^ = 0.09).

## 4. Discussion

Lung cancer survivors remain an understudied subgroup, despite the improvements in their survival rates observed in recent years [12,13]. Therefore, the main objective of this study was to explore HRQOL among LC survivors through two specific objectives: to compare their QoL with survivors of other types of cancer and to explore the possible modulating role of sociodemographic, psychological, social, and clinical variables.

Determining the HRQOL of LC survivors in absolute and relative terms requires a differentiated study of those undergoing treatment versus those in follow-up and those with active disease versus those who are disease-free. The historically low survival rate for LC may have been responsible for grouping in studies participants in different situations and with different problems. However, the notable improvement in survival rates in recent times warrants a more detailed analysis of HRQOL in LC.

Based on a definition of survivor as a person who has completed primary cancer treatment with curative intent and is free of disease, the results of this study support a significant deterioration in the overall HRQOL of LC survivors, placing them alongside survivors with the lowest HRQOL: hematological, breast, and gynecological cancers [15]. The differences and similarities identified cannot be attributed to cohort effects or recruitment procedures, given the homogeneity among survivors of different types of cancer examined. The sociodemographic variables frequently associated in the literature with poorer HRQOL—such as younger age and female sex [3,15,46]—do not appear to account for the decline in HRQOL among lung cancer survivors either. This type of cancer affects both men and women and is characterized by a higher average age at diagnosis than other types of cancer (hematologic, breast, prostate, colorectal, etc.) [47]. Rather, it seems necessary to consider clinical variables such as the high burden of symptoms commonly associated with LC [13,19,48] and the low survival rate that has come to characterize it [12] as factors responsible for the decline in HRQOL experienced by LC survivors.

Despite this, analysis of specific domains of HRQOL reveals some interesting aspects that highlight differences in HRQOL between LC survivors and other survivors. Differences were observed across almost all HRQOL domains. Overall, they suggested a more impaired profile in subgroups with the best HRQOL (prostate and melanoma), and a less impaired profile in subgroups with the lowest HRQOL (hematological and breast). For example, the results of this study did not support the assumption that two common side effects of cancer treatments, such as fatigue and pain, are more severe in LC survivors [9,10,11]. Although LC survivors reported these symptoms to a greater extent than prostate and melanoma survivors, they did not differ from the other subgroups, with the sole exception of the breast cancer subgroup, in which survivors reported experiencing more pain. Similarly, they also reported, to a lesser extent (compared to breast and hematological cancer), another common sequela of cancer treatments: cognitive problems [2].

Differences in treatment may be responsible for the different domains of HRQOL in which hematologic and breast cancer survivors show greater impairment than LC survivors. Standard treatment options for hematologic cancer with high-dose chemotherapy or hematopoietic stem cell transplantation are associated with a high deterioration in HRQOL in this subgroup [49]. In the case of breast cancer, it should be remembered that standard treatment often includes adjuvant hormone therapy as maintenance therapy. The long period for which it is prescribed, as well as the high incidence and severity of its side effects [50], hinders the emotional and physical recovery of breast cancer survivors, even in the long-term survival phase [51].

Finally, it is worth noting the striking result found regarding distress about the possibility of a family member developing cancer. LC survivors experienced more distress compared to survivors of the two types of cancer for which there appears to be greater social awareness of a possible genetic basis: breast cancer and colorectal cancer [15]. Concern about the possibility of a family member developing cancer is a common outcome among cancer survivors [1,3,15] following their first-hand experience of the serious and prolonged impact of cancer diagnosis and treatment. The perception of risk, previously theoretical, becomes real, increasing the perception of vulnerability also for family members [52]. Although we lack a fully satisfactory explanation, it cannot be ruled out that, in the case of LC—even though it is not typically associated with a genetic component—the perception of vulnerability due to the greater knowledge (direct or indirect) that the individual may have acquired through their experience with the disease. Knowledge that would include the observation that their family members may share the same personal and/or environmental risks (family history of heavy smoking, second-hand smoke exposure, or shared socioeconomic environments) that contributed to their own illness.

With regard to the second objective, the results supported the association between distress and poorer HRQOL across all domains assessed by the QLACS. This result is consistent with those obtained in LC [25,53,54] as well as those that have addressed the role of distress as a predictor of HRQOL in cancer survivors in general [21]. Therefore, it seems necessary to integrate routine mental health screening and interventions into oncology care. The detection of clinically significant levels of distress and the exploration of the main associated aspects will enable the implementation of appropriate interventions to improve the HRQOL of cancer survivors. The need to integrate this type of intervention into oncology care is underscored by recent findings supporting the role of distress, partially attributable to elevated levels of systemic inflammation, as an independent predictor of mortality risk in cancer [55,56].

Regarding the third objective, the data supported the role of age, sex, proactive coping, and PSI as independent predictors of HRQOL in lung cancer survivors, accounting for 20% of its variance. In various combinations, the same variables were independent predictors (explained variance ranging from 8% to 23%) of the different dimensions of HRQOL, with the exception of sexual problems and family-related distress.

Although inconsistent results have also been reported [21] common clinical modulators of HRQOL among survivors of other types of cancer, like the type of treatment and the time since its completion [15,36,46,57,58,59] did not influence participants’ HRQOL in this study. Our results are thus consistent with those in the literature that do not support the type of treatment [8,9] or survival length as modulators of HRQOL in LC survivors [9,19,22,54]. If symptoms resulting from damage to a vital organ such as the lung can increase over time [20,60] and cause a permanent reduction in HRQOL [61], it seems reasonable that the impact of treatment and time of survival should be smaller, in accordance with the results obtained by Hussain et al. [19]. The fact that the organ affected in this subgroup of survivors is a vital organ such as the lung may be making the difference. In this regard, it has been noted that (i) the side effects of surgical resection or radiotherapy can be as severe as those associated with systemic treatment [62] and that (ii) symptoms (long-term pulmonary function, dyspnea, pain) resulting from damage (caused by the disease or its treatments) to the lung can increase over time [20,60].

Sociodemographic variables had a greater impact, with age and sex acting as independent predictors of HRQOL. In this sense, consistently with the results obtained in survivors of other types of cancer [3,15,46,63], our results supported being female and being younger as risk factors for poorer HRQOL and are consistent with those who find these same results in LC [8,17,26,27]. In particular, a younger age was associated with more negative feelings and cognitive, financial, and appearance-related problems, and was the only independent predictor of distress—recurrence. It seems clear that the lesser experience and life trajectory of the individual, combined with the greater disruption to their personal and professional development, justifies the greater impact of LC on the HRQOL of younger people. In this regard, it should also be noted that, according to Weiner’s attribution theory [64], part of the impact of lung cancer on the HRQOL of older survivors can be perceived/attributed to aging-related deterioration [52].

Sex was found to be an independent predictor of domains related to cognitive problems, fatigue, and concerns about appearance. Recent results rule out that the differences in HRQOL found between male and female cancer survivors, recent results rule out that they are simply due to the heterogeneity of the groups compared in other sociodemographic and/or disease-related variables (under review). The justification would rather be found in the different biological, psychological, and social profiles that characterize women and men. Specifically, (i) broad sex-based differences in side effects across multiple treatment modalities [65], (ii) both greater neuroticism [66] and a greater tendency to adopt emotional coping strategies, expressing and processing their emotions more openly in women [67,68,69], and (iii) the central role that women continue to assume in home and family care—despite societal changes—which may be significantly challenged by the experience of cancer [70,71,72].

Greater proactive coping was independently associated with fewer negative feelings, fewer cognitive problems, less social avoidance, and more positive feelings. The examination of this type of coping not has been explored in cancer. However, since it refers to the strategies people employ to prevent future stressors [41], proactive coping can be an essential management strategy in contexts where people report reduced HRQOL and have to adapt their lives to the long-term consequences of illness [73].

In line with recent findings in cancer patients [74], correlational results support the association between different types of social support and different domains of HRQOL in LC survivors. Tangible or instrumental support (related exclusively to economic problems) played the smallest role, which is consistent with evidence that this type of support may sometimes reflect the greater burden of disease and, consequently, does not uniformly increase HRQOL [75]. Emotional, informative, PSI and affective support were linked, to a greater or lesser extent, with higher levels of HRQOL in domains related to psychological (positive and negative affectivity), physical (pain, fatigue, cognitive problems), and social (financial problems, social avoidance) areas. On the other hand, however, the strong association found between the different types of social support suggests that LC survivors perceive them as forms of support that are not truly distinguishable from one another. This undifferentiated perception might be the result of the multiples physical and psychosocial challenges cancer survivors face in adapting to their new situation [21,76,77], which require support. In any case, the strong association between them limited the joint analysis of the different types of support as potential independent predictors of HRQOL. Since the results supported a greater role for the PSI, this was the type of support that was explored. PSI was an independent predictor across a large number of HRQOL dimensions: negative emotions, positive emotions, cognitive problems, pain (the only predictor), fatigue, social avoidance, and financial problems. Therefore, it seems that, along with support characterized by the transmission of reassurance, encouragement, affection, and open discussion of concerns related to the disease, having people with whom to spend quality time, have fun, relax, and forget problems proved to be important for better HRQOL in LC survivors. The pivotal role of this type of support is supported by various psychological frameworks such as coping theory [78], behavioural activation theory [79], and Fredrickson’s broaden-and-build theory of positive emotions [80], the theory of positive emotion amplification and construction.

Based on the results obtained regarding the importance of the two psychosocial factors examined on the HRQOL of LC survivors, it is necessary for healthcare teams involved in their direct care delivery to develop programs and interventions that ensure the personal and social well-being of survivors. Competencies that facilitate future-oriented coping can be improved by a brief educational program [81,82]. Consequently, the design of interventions adapted to the cancer context could prepare the LC survivor to cope with the changes that comprise this period of his/her life. Furthermore, based on our findings, it is important to convey to LC survivors the significant positive effects on their HRQOL of engaging in recreational activities that allow them to relax, have fun, take their minds off things, and, ultimately, enjoy themselves. It is also important for healthcare teams to identify any potential gaps in support within the survivor’s social environment so that these gaps can be compensated for through an organizational or institutional framework. Given that the very challenges for which survivors need their support network have the potential to deteriorate the quality of their relationships with others [83], attention to the support network becomes even more important.

One of the strengths of this study was the analysis of HRQOL in LC survivors who are truly in the survival phase of the disease, excluding those with active disease and comparing it with that of survivors of other types of cancer who also meet these criteria. We consider that another strength of the study was the use of a standardized instrument and specially designed for the assessment of HRQOL in cancer survival, which facilitates the comparison and progress of the research results. Nevertheless, several limitations should be noted. Thus, the small size resulting in some of the subgroups based on the variables explored as possible modulators of HRQOL may have influenced the obtaining of significant results. Another limitation of our study was the lack of data on the type of surgical resection the participants underwent, which is closely linked to the examination of the primary treatment’s impact on HRQOL based exclusively on the distinction between local and systemic treatment. Given the wide variations that may exist in the extent of surgical resection (lobectomy, segmentectomy, wedge resection, pneumonectomy), which directly impacts HRQOL, future studies addressing this issue would be of interest. Finally, it is worth noting the cross-sectional nature of the study, which provides only correlational evidence and limits the drawing of conclusions about the relationships’ directionality. Adopting a longitudinal design in future research would allow for a more in-depth exploration of this issue in some of the resulting subgroups.

## 5. Conclusions

The results of this study enhance our understanding of the HRQOL that characterizes the survival phase in lung cancer by defining a survivor as a person who has completed primary treatment with curative intent and is disease-free. The decline in HRQOL experienced by LC survivors is (ii) greater than that reported by survivors of colorectal, head and neck, prostate, and melanoma cancers, (iii) similar to that experienced by survivors of hematologic, breast, and gynecologic cancers, and (iii) closely associated with clinically significant distress. The analysis of potential sociodemographic, clinical, and psychosocial modulators of lung cancer survivors’ HRQOL not only allows for the early identification of those survivors at higher risk of HRQOL decline—who should be prioritized for care—but also demonstrates the potential for improving it. While age and sex cannot be changed, it is possible to develop and implement interventions focused on alleviating distress, improving proactive coping skills, and increasing the social support perceived by lung cancer survivors.

## Figures and Tables

**Table 1:** Characteristics of the participants (N = 141).

Variable	Categories	*n*	%
Age			
Mean: 63.7; SD = 11.0; (Range 23–90)	18–45 years	5	3.6
	46–65 years	63	45.7
	>65 years	70	50.7
Sex	Female	47	33.3
	Male	94	66.7
Marital status	With Partner	104	74.3
	Single	36	25.7
Level of studies	Below High school	55	40.5
	High school	32	23.5
	Above high school	49	36.0
Employment status	Employed (active + homemaker + students)	30	21.6
	Non employed (retired, disabled, unemployed)	109	78.4
Primary treatments	Local	26	18.4
	Systemic	115	81.6
Survival phase (months) Mean: 35.9; SD = 47.1; (Range = 1–336)	RES	40	31.3
	ES	56	43.4
	LTS	32	25.0

Note. RES, re-entry survivorship (≤12); ES, early survivorship (13–59); LTS, long-term survivorship (≥60).

**Table 2:** Differences by cancer type (Pillai’s Trace = 0.403; F = 8.97; df hyp = 77.00; df error = 11,312.00; *P* = 0.000).

QLACS Domains	Lung	H	B	G	C	H/N	M	*P*
Negative feelings (F = 15.31 ***)	12.01 ^h,p^ (4.81)	14.16 (4.91)	13.26 (5.22)	13.10 (5.40)	11.26 (4.84)	11.47 (4.96)	11.94 (4.58)	10.12 (4.85)
Positive feelings (F = 5.62 ***)	21.33 (5.79)	19.23 (5.21)	19.77 (5.89)	20.25 (6.38)	21.10 (5.98)	21.30 (5.52)	20.23 (5.45)	22.10 (5.32)
Cognitive problems (F = 20.80 ***)	10.34 ^h,b^ (4.80)	13.88 (5.57)	12.41 (5.75)	10.62 (5.62)	10.21 (4.96)	9.56 (4.67)	9.82 (5.09)	8,89 (4.56)
Physical pain (F = 25.71 ***)	10.59 ^b,m,p^ (5.92)	12.39 (6.32)	12.61 (6.32)	12.45 (7.52)	9.68 (5.68)	11.27 (6.07)	8.11 (3.91)	7.84 (4.76)
Fatigue (F = 18.58 ***)	13.34 ^m,p^ (5.72)	14.27 (5.79)	13.82 (5.70)	13.11 (6.44)	11.69 (5.76)	12.11 (5.00)	10.59 (5.11)	9.78 (5.29)
Social Avoidance (F = 5.60 ***)	8.90 ^p^ (6.02)	10.77 (5.13)	8.65 (5.49)	8.45 (5.13)	8.22 (5.24)	7.96 (5.20)	7.89 (4.17)	7.10 (4.89)
Sexual problems (F = 10.73 ***)	11.51 ^h^ (5.96)	13.82 (6.83)	13.06 (6.71)	13.63 (6.73)	11.13 (6.18)	9.36 (4.92)	9.09 (4.83)	12.61 (6.29)
Financial problems (F = 12.96 ***)	6.17 ^h^ (4.25)	9.12 (5.69)	7.37 (5.40)	7.02 (5.21)	6.30 (4.61)	7.24 (5.13)	5.15 (2.39)	5.10 (2.69)
Distress family (F = 9.14 ***)	14.04 ^b,c^ (6.62)	12.56 (5.59)	13.73 (5.85)	13.07 (6.50)	13.67 (5.82)	13.00 (5.46)	12.17 (5.44)	11.18 (6.50)
Appearance concerns (F = 54.89 ***)	8.50 ^h,b,g,p^ (5.82)	13.60 (6.57)	11.82 (6.74)	11.25 (7.10)	7.46 (4.35)	8.22 (5.13)	6.72 (3.08)	5.35 (3.06)
Distress recurrence (F = 17.24 ***)	14.77 ^p^ (7.06)	16.00 (6.35)	15.14 (6.86)	15.25 (7.59)	12.98 (6.29)	14.02 (7.09)	14.32 (6.15)	10.52 (5.85)
Total Scale (F = 31.80 ***)	136.12 ^c,hn,m,p^ (40.37)	152.27 (37.92)	141.70 (37.35)	136.49 (39.90)	123.25 (33.69)	125.87 (30.55)	117.00 (25.22)	110.21 (28.04)

Note. *** *p* < 0.000. Higher numerical scores = lower HRQOL. Significant differences with respect to the ^b^: breast; ^p^: prostate; ^c^: colorectal; ^h^: hematologic; ^hn^: head/neck; ^g^: gynaecologic; ^m^: melanoma.

**Table 3:** Differences by age, sex, and distress caseness.

	Age (Pillai’s Trace = 0.175; F = 2.44; df hyp = 11.00; df error = 126.00; *p* = 0.009)				Sex (Pillai’s Trace = 0.190; F = 2.69; df hyp = 11.00; df error = 126.00; *p* = 0.004)				Distress Caseness (Pillai’s Trace = 0.333; F = 5.72, df hyp = 11.000, df error = 126.000, *p* ≤ 0.001)			
QLACS Domains	18–64 Years (N = 64)	≥65 Years (N = 74)	F (Sig.)	ηp^2^	Female (N = 47)	Male (N = 94)	F (Sig.)	ηp^2^	No Case (N = 100)	Case (N = 41)	F (Sig.)	ηp^2^
Negative feelings	12.92 (4.45)	11.22 (5.00)	4.42 *	0.031	13.40 (4.83)	11.33 (4.68)	5.78 *	0.041	10.56 (3.57)	15.69 (5.60)	41.20 ***	0.233
Positive feelings	20.75 (5.79)	21.82 (5.78)	1.19	0.009	20.44 (5.15)	21.75 (6.05)	1.56	0.011	9.17 (4.82)	14.49 (6.32)	28.34 ***	0.172
Cognitive problems	11.33 (5.04)	9.49 (4.44)	5.21 *	0.037	11.89 (5.14)	9.59 (4.46)	7.27 **	0.051	9.43 (4.53)	12.64 (4.74)	13.66 ***	0.091
Sexual problems	11.73 (5.98)	11.31 (5.98)	0.18	0.001	12.80 (6.07)	10.88 (5.83)	3.19	0.023	10.66 (5.40)	13.67 (6.79)	7.47 **	0.052
Pain	11.77 (5.74)	9.57 (5.93)	4.86 *	0.034	11.64 (5.33)	10.08 (6.15)	2.15	0.016	8.83 (4.57)	15.05 (6.64)	39.57 ***	0.225
Fatigue	13.69 (5.34)	13.04 (6.05)	0.44	0.003	15.02 (5.63)	12.53 (5.61)	5.99 *	0.042	12.20 (5.18)	16.23 (6.05)	15.35 ***	0.101
Social avoidance	9.58 (6.06)	8.31 (5.97)	1.53	0.011	10.16 (6,87)	8.29 (5.50)	2.95	0.021	7.28 (4.51)	13.00 (7.36)	30.69 ***	0.184
Appearance concerns	10.56 (6.71)	6.72 (4.21)	16.74 ***	0.110	11.02 (6.77)	7.28 (4.88)	13.73 ***	0.092	7.52 (4.88)	11.00 (7.18)	10.77 ***	0.073
Financial problems	7.47 (5.11)	5.05 (2.93)	11.98 ***	0.081	6.98 (4.81)	5.78 (3.92)	2.41	0.017	5.38 (2.97)	8.18 (6.05)	13.20 ***	0.088
Distress—recurrence	16.81 (6.91)	13.00 (6.75)	10.72 ***	0.073	16.67 (7.09)	13.85 (6.90)	4.97 *	0.035	13.54 (6.74)	17.90 (6.98)	11.50 ***	0.078
Distress—family	15.03 (6.07)	13.19 (6.99)	2.67	0.019	13.27 (6.03)	14.42 (6.89)	0.92	0.007	13.27 (6.62)	16.02 (6.28)	4.98 *	0.035
Total Scale	148.59 (30.05)	125.32 (39.42)	12.35 ***	0.083	152.89 (39.21)	128.00 (38.56)	12.50 ***	0.084	123.15 (32.13)	169.02 (40.79)	48.71 ***	0.264

Note. *** *p* < 0.000; ** *p* < 0.001; * *p* < 0.05. ηp^2^ = Partial eta squared. Higher numerical scores = lower HRQOL.

**Table 4:** Correlations between HRQOL, Proactive Coping, and Social Support.

	Proactive Coping	Emotional Support	Informational Support	Tangible Support	Positive Social Interaction	Affective Support	Total Support
Emotional support	0.16						
Informational support	0.18 *	0.93 ***					
Tangible support	0.03	0.77 ***	0.75 ***				
Positive social interaction	0.13	0.85 ***	0.80 ***	0.69 ***			
Affective support	0.13	0.76 ***	0.72 ***	0.66 ***	0.84 ***		
Total support	0.13	0.96 ***	0.93 ***	0.86 ***	0.91 ***	0.87 ***	
Negative feelings	−0.28 ***	−0.21 *	−0.17 *	−0.13	−0.23 **	−0.23 **	−0.20 *
Positive feelings	−0.38 ***	−0.34 ***	−0.34 ***	−0.16	−0.34 ***	−0.30 ***	−0.32 ***
Cognitive problems	−0.22 **	−0.23 **	−0.17 *	−0.12	−0.20 *	−0.25 **	−0.20 *
Pain	−0.10	−0.11	−0.12	−0.06	−0.23 **	−0.20 *	−0.13
Sexual problems	−0.05	−0.14	−0.13	−0.17 *	−0.07	−0.06	−0.11
Fatigue	−0.20 *	−0.18 *	−0.22 *	−0.09	−0.21 *	−0.17	−0.18 *
Social avoidance	−0.29 ***	−0.06	−0.06	0.01	−0.20 *	−0.15	−0.08
Financial problems	−0.05	−0.26 **	−0.26 **	−0.23 **	−0.23 **	−0.24 **	−0.25 **
Distress—family	−0.01	0.11	0.11	0.08	0.08	0.09	0.10
Appearance concerns	−0.07	−0.05	−0.11	−0.08	−0.08	−0.12	−0.07
Distress—recurrence	−0.03	−0.07	−0.02	−0.09	−0.09	−0.07	−0.06
Total Scale	−0.19 *	−0.19 *	−0.20 *	−0.12	−0.25 **	−0.22 **	−0.20 *

Note. *** *p* < 0.000; ** *p* < 0.001; * *p* < 0.05.

**Table 5:** Predictors of overall HRQOL and by domain.

			Age			Sex			Proactive Coping			PSI		
	F (df1,df2)	Adjusted R^2^	β	t	*p*	β	t	*p*	β	t	*p*	β	t	*p*
Negative feelings	6.706 (4,133) ***	0.143	−0.17	−2.11	0.037	−0.126	−1.55	0.123	−0.25	−3.08	0.003	−0.18	−2.25	0.026
Positive feelings	21.129 (2,135) ***	0.23							−0.35	−4.61	<0.001	−0.30	−3.91	<0.001
Cognitive problems	6.151 (4,133) ***	0.131	−0.19	−2.35	0.020	−0.17	−2.08	0.039	−0.20	−2.41	0.017	−0.15	−1.84	0.068
Fatigue	5.278 (3,134) ***	0.086				−0.18	−2.15	0.033	−0.16	−1.89	0.061	−0.17	−2.03	0.045
Social avoidance	8.896 (2,135) ***	0.103							−0.28	−3.38	0.001	−0.17	−2.04	0.044
Financial problems	9.307 (2,136) ***	0.107	−0.26	−3.24	0.001							−0.22	−2.76	0.007
Appearance concerns	13.467 (2,138) ***	0.151	−0.26	−3.32	0.001	−0.268	−3.32	0.001						
Distress—recurrence	7.849 (2,138) ***	0.089	−0.25	−3.10	0.002	−0.16	−1.94	0.054						
Total Scale	9.46 (4,131) ***	0.20	−0.27	−3.41	0.001	−0.21	−2.66	0.009	−0.17	−2.14	0.034	−0.21	−2.68	0.008

Note. PSI = Positive Social Interaction, *** *p* < 0.000.

## Data Availability

The datasets generated during and/or analysed during the current study are available from the corresponding author on reasonable request.
